# Study on the therapeutic effect of eggshell membrane on osteoarthritis in rats

**DOI:** 10.1371/journal.pone.0346166

**Published:** 2026-04-03

**Authors:** Mingming Pan, Yanhua Shen, Jiayu Wu, Chaonan Liu, Meihong Zhu, Zhengyu Zhou

**Affiliations:** 1 Laboratory Animal Center of Suzhou Medical College, Soochow University, Suzhou, Jiangsu, China; 2 HS NUTRA CO., LTD., Zhejiang, China; Dow University of Health Sciences, PAKISTAN

## Abstract

This study aimed to investigate the therapeutic effects of ELASEM^®^Flex and ELASEM^®^ProFlex, two eggshell membrane (EM) products, on sodium iodoacetate (MIA)-induced osteoarthritis (OA) in rats. An OA model was established by a single intra-articular injection of MIA into the knee joint. After modeling, rats were administered diclofenac sodium, ELASEM^®^Flex, and ELASEM^®^ProFlex by gavage daily for 4 consecutive weeks. During the experiment, food intake, water intake, body weight, and plantar mechanical pain threshold (MPT) of rats were measured weekly. Serum levels of TNF-α, COX-2, IL-1β, and CTX-II were assessed at weeks 2 and 4. After 4 weeks, knee joints were harvested for histopathological examination (HE staining and Safranin-O fast green staining). Results indicated that knee joints of OA rats showed significant swelling, which was alleviated to varying degrees in all treatment groups. Both ELASEM^®^Flex and ELASEM^®^ProFlex significantly increased the MPT (P < 0.05) and demonstrated sustained analgesic effects. These treatments also significantly reduced the serum levels of IL-1β, COX-2, TNF-α, and CTX-II at weeks 2 and 4 (P < 0.05). Histopathological analysis revealed that both EM preparations markedly alleviated arthritis symptoms, improved cartilage structure, promoted chondrocyte proliferation, enhanced staining of chondrocytes and cartilage matrix, and resulted in significantly lower Mankin's scores compared to the OA model group (P < 0.05). These results indicate that ELASEM^®^Flex and ELASEM^®^ProFlex can exert preventive and reparative effects on knee OA in rats by alleviating arthritis pain, inhibiting inflammatory factor expression, reducing type II collagen degradation, and promoting chondrocyte proliferation.

## Introduction

OA, a common degenerative joint disease [1–2], is characterized by articular cartilage degeneration, synovial inflammation, and subchondral bone remodeling, ultimately leading to joint dysfunction and pain [3–5]. Global burden of disease data indicated a rising number of OA cases, reaching 41.468 million by 2019, with China having the highest prevalence, particularly among individuals aged 40 years and above (46.3%) [6]. As the population ages, OA has become a serious public health issue, not only reducing patients’ quality of life but also imposing a substantial socioeconomic burden [7–9]. Current clinical treatments for OA include nonsteroidal anti-inflammatory drugs (NSAIDs), corticosteroids, hyaluronic acid intra-articular injections, and surgical approaches [10]. However, long-term NSAIDs use may cause gastrointestinal and cardiovascular side effects, while repeated intra-articular injections of corticosteroids are associated with cartilage loss [11–13]. Surgical treatments, such as arthroscopic debridement and total knee arthroplasty, can effectively alleviate symptoms, but are associated with risks including infection, thrombosis, and subsequent prosthesis loosening [14]. Therefore, developing safe and effective natural alternative therapies has gained research interest [15–17]. EM, a by-product of egg processing, contains major active components such as collagen, hyaluronic acid, chondroitin sulfate, and glycosaminoglycans, which are similar to the components of joint synovium and cartilage matrix [18]. Previous studies have shown that EM can significantly improve joint swelling and function in OA rats by inhibiting the release of inflammatory factors and promoting cartilage matrix synthesis [11,18,19]. ELASEM^®^Flex and ELASEM^®^ProFlex are novel EM products developed for joint health. Their raw materials are sourced from the high-purity EM of fresh poultry eggs, which contains collagen (types I, III, IV, V, VII, VIII, X, XII, and XXII), hyaluronic acid, chondroitin sulfate, and other natural ingredients. Compared to ELASEM^®^Flex, ELASEM^®^ProFlex has a higher degree of hydrolysis, and exhibits better water solubility and greater bioavailability. This study utilized a MIA-induced rat OA model to evaluate the therapeutic effects of ELASEM^®^Flex and ELASEM^®^ProFlex across three dimensions: pain relief, inflammation inhibition, and cartilage protection.

## Materials and methods

### Ethics statement

All animal experiments complied with the Management Measures for Laboratory Animals of Soochow University, and the protocol was approved by the Ethics Committee of Soochow University (protocol number:202409A258).

### Animals and housing

Forty male SPF-grade Sprague-Dawley (SD) rats, weighing 180–220 g, were provided by Vital River Laboratory Animal Technology Co., Ltd, Zhejiang, China. The animals were housed in the facility of the Laboratory Animal Center of Soochow University and acclimatized for one week under controlled conditions: temperature 20–26°C, humidity40%−70%, and a 12 h light-dark cycle. Rats had free access to standard rodent chow and water.

### Osteoarthritis rodent animal model and treatment

Forty rats were randomly divided into five groups: normal control (A), OA model (B), positive control (C), ELASEM^®^Flex (D), and ELASEM^®^ProFlex (E). Rats were anesthetized via intraperitoneal injection of a combination of Stesil (Virbac S.A., France) and xylazine hydrochloride (Huamu Animal, Jilin, China) to eliminate procedural pain. The right hind knee was shaved and disinfected with 75% alcohol. With the knee joint flexed at 90°, a 1 mL syringe needle was inserted along the patellar ligament toward the femoral condyle. A distinct puncture sensation indicated entry into the joint cavity [20], and the drug was injected slowly. Groups B- E received 50 μL of 2 mg MIA (Yuanye Biotechnology, Shanghai, China) solution to induce OA in the right hind limb, group A received 50 μL normal saline. After modeling, groups C, D, and E were received daily gavage of diclofenac sodium (2 mg/kg/day), ELASEM^®^Flex (31.5 mg/kg/day, human equivalent dose of 300 mg/60 kg/day), or ELASEM^®^ProFlex (31.5 mg/kg/day, human equivalent dose of 300 mg/60 kg/day), respectively [21,22]. All products were manufactured and provided by HS NUTRA CO., LTD., Zhejiang, China. Groups A and B received equivalent volumes of normal saline for 4 consecutive weeks. After 4 weeks of administration, rats were euthanized by the carbon dioxide method (RWD Life Science, Shenzhen, China). Throughout the experiment, efforts were made to minimize animal suffering, including optimized housing conditions, standardized handling, and continuous monitoring of animal well-being.

### Measurement of body weight, food intake and water intake

During the experiment, body weight, food intake, and water intake of rats were measured weekly.

### Detection of MPT

The MPT was measured pre-modeling (day 0) and on days 7, 14, 21, and 28 post-modeling using a Bioseb Bio-EVF5 algesimeter (Bioseb, France). Rats were placed in a transparent grid box and acclimatized for 30 min to minimize animal stress. The algesimeter needle was applied vertically to the right hind paw with gradual pressure. The value at which the rat withdrew or licked the paw was recorded as the threshold. Measurements were taken in triplicate with 5-min intervals between tests [23].

### Measurement of TNF-α, COX-2, IL-1β, and CTX-II level using assay kits

After animals were anesthetized with isoflurane, blood was collected via vein puncture at weeks 2 and 4. Serum was separated by low-temperature centrifugation (Eppendorf, Germany), and TNF-α, COX-2, IL-1β, and CTX-II levels were measured using ELISA kits (Enzyme-linked, Shanghai, China).

### Histopathological analysis

After rats were euthanized, the right hind knee joint was quickly removed and fixed in 4% paraformaldehyde (Labgic, Beijing, China) for 48 h, then transferred to EDTA solution (Solarbio, Beijing, China) for decalcification. After complete decalcification, the tissues were dehydrated with gradient ethanol, cleared with xylene, and then embedded in paraffin. Continuous sections of the paraffin blocks were cut with a microtome (Leica Microsystems, Germany) at a thickness of approximately 5 μm. The sections were subjected to HE staining and Safranin-O fast green staining, and finally mounted with neutral gum for microscopic examination (Leica Microsystems, Germany). Pathological sections of each group were scored according to the Mankin’s scoring criteria. The scoring categories consisted of cartilage structure (score: 0–6), chondrocyte cellularity (score: 0–3), cartilage matrix Safranin-O staining intensity (score: 0–4), and tidemark integrity (score: 0–1). The total score of this system ranges from 0 to 14, with a higher score indicating a more severe degree of articular cartilage degeneration and OA progression [18,24–26].

### Statistical analysis

All data were analyzed using SPSS 20 software (IBM, Armonk, NY, USA). Results were expressed as mean ± standard deviation (x ± SD). Normality of the data distribution was assessed using the Shapiro-Wilk test, and homogeneity of variances was evaluated using Levene’s test. For measured data that satisfied the assumptions of normality and homogeneity of variances, one-way analysis of variance (ANOVA) was employed, followed by Tukey's HSD post hoc test for multiple comparisons. For scored parameters and data that violated the assumption of normality, the non-parametric Kruskal-Wallis test was applied, followed by Dunn’s test with Bonferroni correction for post-hoc pairwise comparisons. A P value < 0.05 was considered statistically significant.

## Results

### Knee joint and activity status

All OA rats exhibited arthritis symptoms post-modeling: joint swelling, stiffness, and limping. Swelling was evident in model rats, with varying degrees of relief in treatment groups.

### Effects of EM on body weight, food intake and water intake

**Fig 1:**
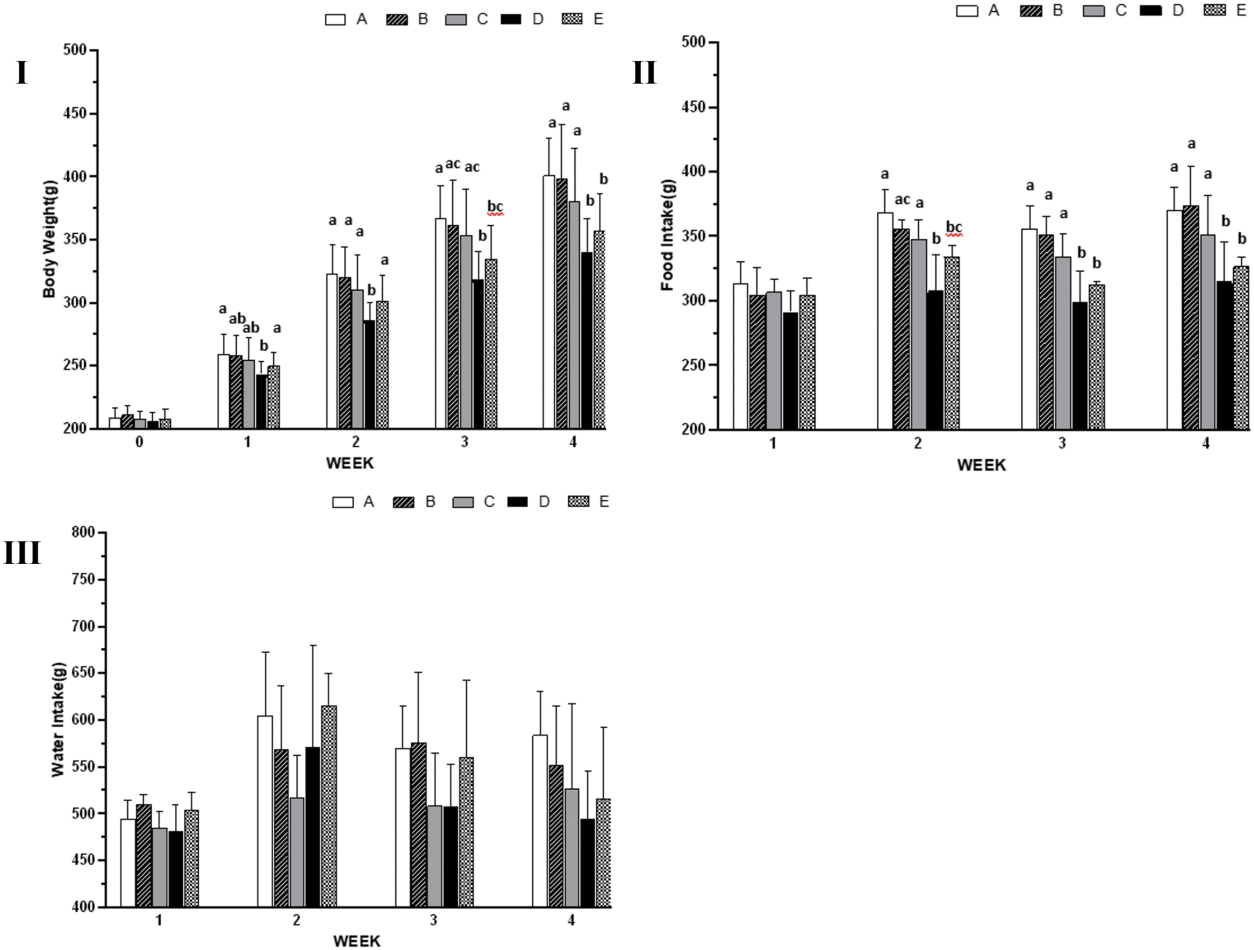
Effects of EM on body weight, food intake and water intake. **(I)** Body weight changes at different weeks post-modeling. **(II)** Food intake changes at different weeks post-modeling. **(III)** Water intake changes at different weeks post-modeling. Values are expressed as mean ± SD of 8 rats. A: normal control group; B: OA model control group; C: positive control group; D: ELASEM^®^Flex group; E: ELASEM^®^ProFlex group. Bars with different letters indicate that they were statistically different at P < 0.05.

Compared to groups A and B, groups D and E showed reduced body weight and total weight gain at weeks 1–4 (Fig 1I); the food intake in groups D and E at 2–4 weeks and total food intake were significantly reduced (Fig 1II); there was no significant difference in water intake among all groups compared with groups A and B (Fig 1III).

### Effects of EM on MPT

**Fig 2:**
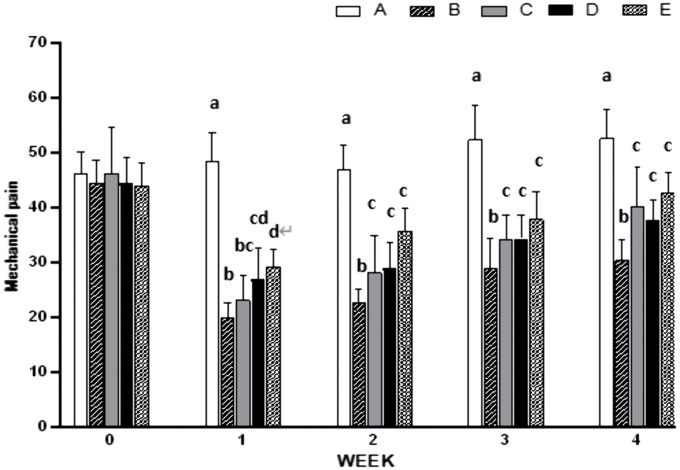
Weekly effects of EM on MPT in rats after modeling. Values are expressed as mean ± SD of 8 rats. A: normal control group; B: OA model control group; C: positive control group; D: ELASEM^®^Flex group; E: ELASEM^®^ProFlex group. Bars with different letters indicate that they were statistically different at P < 0.05.

The results showed that compared with the normal control group (A), the MPT in the OA model group (B) was significantly decreased from week 1 to week 4 after modeling (P < 0.05). Compared to group B, the MPT of the positive control group (C) was significantly increased at weeks 2–4 (P < 0.05), by 28%, 19%, and 32% respectively; the MPT of group D (ELASEM^®^Flex) was significantly increased at weeks 1–4 (P < 0.05), by 35%, 28%, 19%, and 24% respectively; the MPT of group E(ELASEM^®^ProFlex) was significantly increased at 1–4 weeks (P < 0.05) by 46%, 59%, 30%, and 40% respectively. Compared to group C, group D showed no significant difference at weeks 1–4 (P > 0.05), while group E showed significant increases at weeks 1 (P < 0.05) and non-significant increases at weeks 2–4 (P > 0.05), by 21%, 24%, 10%, and 6% respectively. Groups C, D, and E showed a decrease in threshold at week 1 post-modeling but an upward trend thereafter. Group E’s threshold at week 4 was closest to group A but still significantly different (Fig 2).

### Effects of EM on serum levels of IL-1β, COX-2, and TNF-α

**Fig 3:**
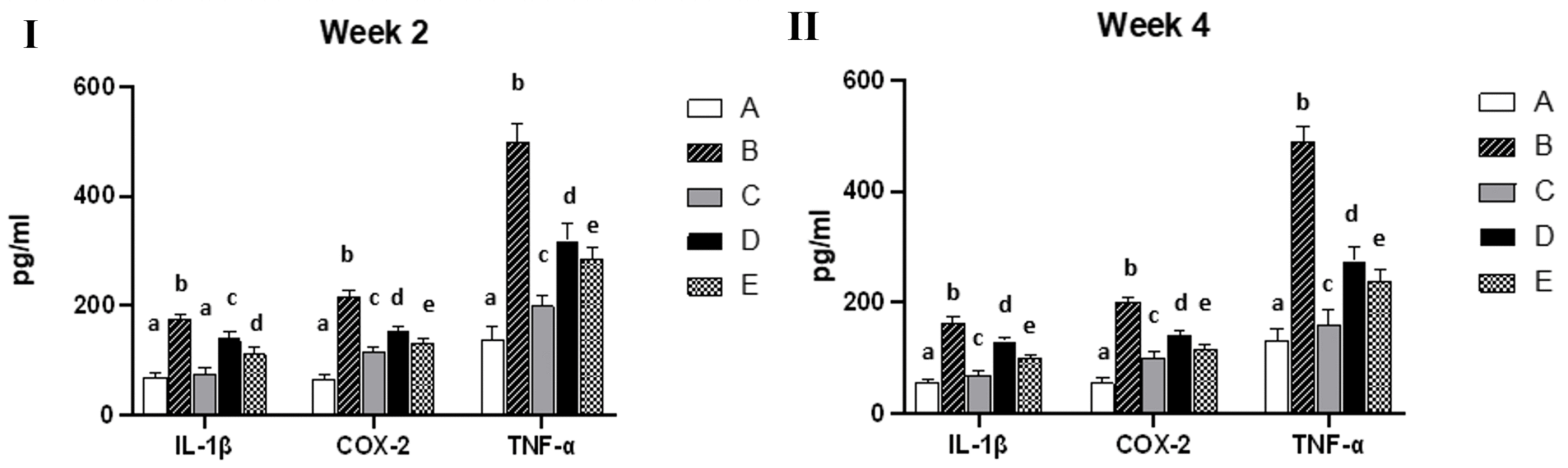
Effects on IL-1β, COX-2, and TNF-α levels at weeks 2 and 4 after modeling. **(I)** IL-1β, COX-2, and TNF-α levels at weeks 2 after modeling; **(II)** IL-1β, COX-2, and TNF-α levels at weeks 4 after modeling. Values are expressed as mean ± SD of 8 rats. A: normal control group; B: OA model control group; C: positive control group; D: ELASEM^®^Flex group; E: ELASEM^®^ProFlex group. Bars with different letters indicate that they were statistically different at P < 0.05.

Serum levels of IL-1β, COX-2, and TNF-α were significantly elevated in group B at weeks 2 and 4 compared to group A (P < 0.05). Compared to the OA model group (B), the serum levels of IL-1β, COX-2, and TNF-α in the positive control group (C) were significantly decreased at weeks 2 and 4 (P < 0.05); the serum levels of IL-1β, COX-2, and TNF-α in the ELASEM^®^Flex group (D) were significantly decreased at weeks 2 and 4 (P < 0.05) by 20%, 28%, and 36% at week 2, and 21%, 29%, and 44% at week 4, respectively. The serum levels of IL-1β, COX-2, and TNF-α in the ELASEM^®^ProFlex group (E) were significantly decreased at weeks 2 and 4 (P < 0.05) by 37%, 39%, and 43% at week 2, and 39%, 42%, and 51% at week 4, respectively (Fig 3).

### Effects of EM on CTX-II

**Fig 4:**
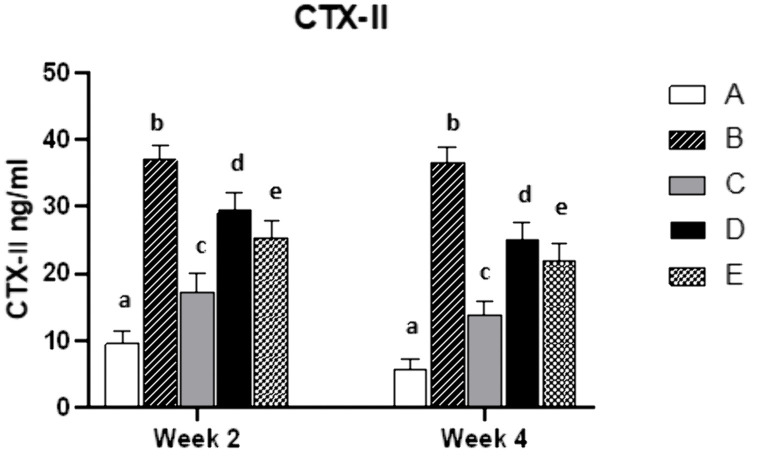
Effects of EM on CTX-II levels at weeks 2 and 4 post-modeling. Values are expressed as mean ± SD of 8 rats. A: normal control group; B: OA model control group; C: positive control group; D: ELASEM^®^Flex group; E: ELASEM^®^ProFlex group. Bars with different letters indicate that they were statistically different at P < 0.05.

The CTX-II levels were significantly higher in group B than in group A at weeks 2 and 4 (P < 0.05). Compared to the OA model group (B), Group C reduced CTX-II by 54% and 62% at weeks 2 and 4, respectively (P < 0.05). Group D reduced it by 20% and 32%, and group E by 32% and 40% (P < 0.05) (Fig 4).

### Histopathological examination results

**Fig 5:**
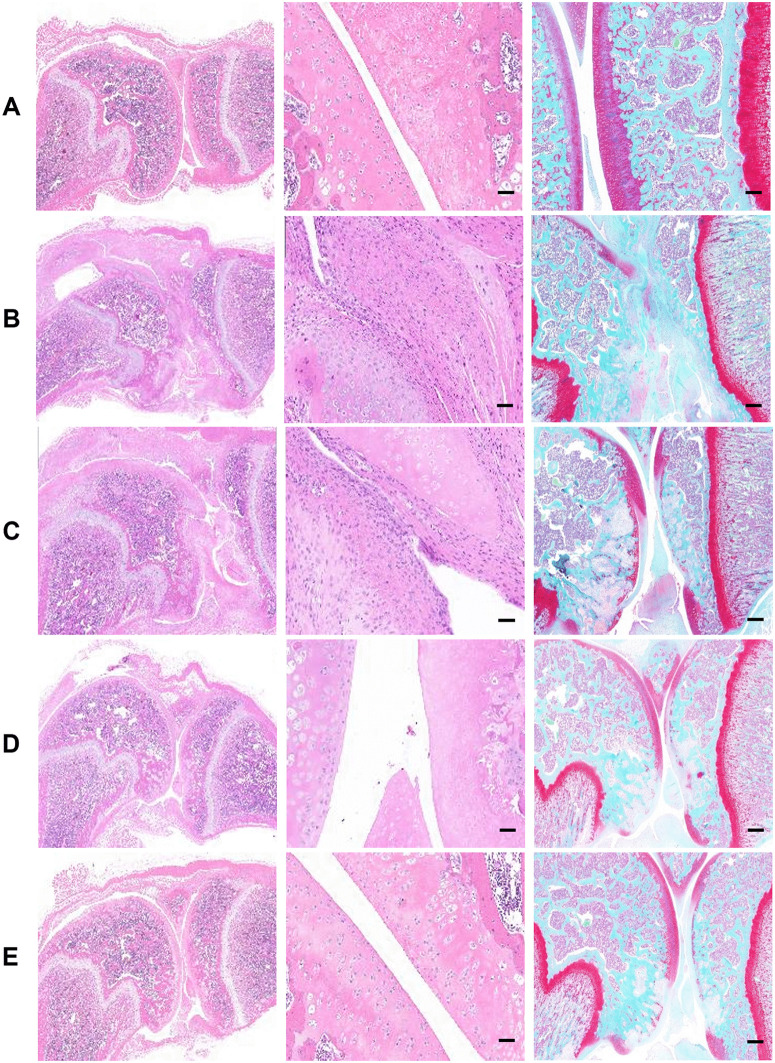
Histopathological examination results of rats in each group. A: normal control group; B: OA model control group; C: positive control group; D: ELASEM^®^Flex group; E: ELASEM^®^ProFlex group. The three images in each group are the local panorama of HE staining, microscopic results of HE staining (200×), and Safranin-O fast green staining (50×). The scale bar for 200 × magnification is 50 μm, and 200 μm for 50 × magnification.

HE and Safranin-O fast green staining results showed that compared with the normal control group, group B exhibited severe arthritis: thinning of hyaline cartilage, chondrocyte degeneration and necrosis, reduced cell count, pale staining of cells and matrix, local unstained areas, disrupted tide line, fibrous tissue proliferation, narrowed joint space, synovial congestion and thickening, subchondral bone involvement, and meniscal and ligament damage. After treatments with diclofenac sodium, ELASEM^®^Flex, and ELASEM^®^ProFlex, the thinned areas of hyaline cartilage on the knee joint surface were reduced, chondrocyte proliferation and number were increased, the narrowing of the joint space was improved, the congested and thickened areas of synovial tissue were reduced, the subchondral bone tissue was improved, and the staining of chondrocytes and cartilage matrix was increased compared with the OA model group (Fig 5).

### Histopathological scoring

The Mankin's score was significantly higher in group B than in group A (P < 0.05). All treatment groups showed significantly lower scores than group B (P < 0.05) (Table 1).

**Table 1:** Mankin’s Score in Each Group.

Group	Histopathological score
Normal control group (A)	0.0 ± 0.0^#^
OA model control group (B)	11.2 ± 1.2
Positive control group (C)	8.3 ± 2.3^#^
ELASEM®Flex group（D）	9.0 ± 1.8^#^
ELASEM^®^ProFlex group（E）	8.8 ± 1.3^#^

Results are expressed as mean ± SD of 8 rats. After Dunn's multiple comparison test with Bonferroni correction: compared with the OA model control group (B), # P < 0.05; compared with the positive control group (C), * P < 0.05.

## Discussion

OA is the most common joint disease in the elderly, affecting nearly 70% of individuals during their lifetime, and imposing significant economic and social burdens on patients and healthcare systems [1,4,5]. With the global aging population and increasing obesity rates, OA incidence is rising year by year. Current treatments alleviate symptoms but do not halt disease progression [27], and drug often have side effects. Therefore, safe and effective alternative therapies are needed. EM, known as “Phoenix Cloth” in traditional Chinese medicine, is rich in fibrin, collagen, hyaluronic acid, chondroitin sulfate, and glucosamine [19]. Recent studies indicate that EM has anti-inflammatory properties and is similar to synovial components, making it a potential alternative drug or functional food ingredient that can exert anti-inflammatory and cartilage-protective effects in the treatment of OA [19], supported by clinical trials and animal studies [28–34]. This study used an MIA-induced rat OA model to systematically evaluate ELASEM^®^Flex and ELASEM^®^ProFlex. MIA inhibits glyceraldehyde-3-phosphate dehydrogenase, causing cartilage degradation, loss, and degenerative changes of the cartilage matrix, similar to human OA [20]. Diclofenac sodium, a COX inhibitor belonging to NSAIDs, is widely used in the treatment of OA [12]. In this study, diclofenac sodium was used as a positive control to compare the therapeutic effects of the two EM preparations, ELASEM^®^Flex and ELASEM^®^ProFlex.

In this experiment, OA rats exhibited obvious arthritis symptoms after modeling: joints swelling, joint stiffness and limping. Compared to the normal control group (A), Group B showed decreased MPT (P < 0.05) and increased serum IL-1β, COX-2, TNF-α, and CTX-II (P < 0.05); Histopathology confirmed severe cartilage damage and higher Mankin's scores in group B (P < 0.05). These results fully confirmed the successful establishment of the OA rat model in this study. During the experiment, the body weight and total weight gain of rats in the groups ELASEM^®^Flex (D)and ELASEM^®^ProFlex at weeks 2–4 were decreased to varying degrees compared to group B which may be due to the anti-obesity effect of EM [35].

Pain relief is a key OA treatment goal [12]. Both ELASEM^®^Flex and ELASEM^®^ProFlex significantly increased the MPT of OA rats. ELASEM^®^Flex increased the MPT of OA rats by 35%, 28%, 19%, and 24% at 1–4 weeks, respectively, while ELASEM^®^ProFlex increased it by 46%, 59%, 30%, and 40%, respectively, indicating that ELASEM^®^Flex and ELASEM^®^ProFlex can alleviate arthritis pain to a certain extent with a sustained analgesic effect. Notably, ELASEM^®^ProFlex exhibited analgesic activity as early as 7 days after administration, with an onset time earlier than the positive control drug diclofenac sodium. Compared with the diclofenac sodium group, there was no significant difference in the MPT of the ELASEM^®^Flex group (D) at weeks 1–4 (P > 0.05), while the MPT of the ELASEM^®^ProFlex group (E) was significantly increased at weeks 1 (P < 0.05) and showed an upward trend at weeks 2–4 without statistical significance (P > 0.05), with increases of 21%, 24%, 10%, and 6% respectively. These results indicate that the analgesic effect of ELASEM^®^ProFlex is significantly better than that of diclofenac sodium, while the analgesic effect of ELASEM^®^Flex is comparable to that of diclofenac sodium.

Proinflammatory cytokine such as interleukin-1β (IL-1β), tumor necrosis factor-α (TNF-α), and cyclooxygenase-2 (COX-2) play pivotal roles in the pathogenesis of OA and are recognized as key biomarkers for the disease [36–39]. IL-1β, a proinflammatory cytokine, can activate other immune cells, stimulate the release of inflammatory mediators, and induce inflammatory symptoms such as fever and pain. Its levels are frequently elevated in the progression of conditions such as arthritis and cardiovascular diseases. Additionally, IL-1βpromotes the secretion of matrix metalloproteinases, which contribute to cartilage matrix degradation and chondrocyte apoptosis. TNF-α is another important proinflammatory cytokine. In arthritic conditions, elevated TNF-α levels can trigger the activation of large number of leukocytes and reduce the synthesis of type II collagen, thereby exacerbating cartilage degradation [40,41]. COX-2, an inducible enzyme, is minimally expressed under normal physiological conditions, but shows significantly upregulated expression upon inflammatory stimulation or cytokine activation [42]. It catalyzes the conversion of arachidonic acid to prostaglandins, participating in pathological processes including inflammatory response, pain, and fever. Consequently, IL-1β, COX-2, and TNF-α represent important targets for OA. The findings of this study demonstrated that ELASEM^®^Flex and ELASEM^®^ProFlex significantly reduced the serum levels of IL-1β, COX-2, and TNF-α. Specifically, ELASEM^®^Flex exhibited inhibition rates of 20%, 28%, and 36% for IL-1β, COX-2, and TNF-α at week 2, respectively, with corresponding rates of 21%, 29%, and 44% at week 4. For ELASEM^®^ProFlex, the inhibition rates at week 2 were 37%, 39%, and 43%, and at week 4 were 39%, 42%, and 51% for the same cytokines. These results indicate that both ELASEM^®^Flex and ELASEM^®^ProFlex can effectively alleviate knee joint inflammation in OA rats by suppressing the expression of proinflammatory cytokines, with ELASEM^®^ProFlex demonstrating a significantly superior effect compared to ELASEM^®^Flex.

The extracellular matrix (ECM) of articular cartilage is primarily composed of collagen and proteoglycans [43–45]. A hallmark feature of OA is the loss of articular cartilage components, which leads to cartilage tissue degradation, reduced cellularity, and ultimately, loss of joint function. Thus, preventing ECM degradation stands as a key objective in anti-OA therapy [46–49]. CTX-II (C-terminal cross-linked peptide of type II collagen) serves as a specific marker for type II collagen degradation. As the main component of articular cartilage, type II collagen undergoes degradation when articular cartilage degenerates or is damaged, releasing CTX-II into the bloodstream or synovial fluid. CTX-II is a critical biomarker for evaluating OA [6], elevated levels directly indicate articular cartilage breakdown and correlate positively with the severity of cartilage damage in conditions such as OA and rheumatoid arthritis (RA) [50,51]. Proinflammatory cytokine (such as TNF-α and IL-1β) can activate the NF-κB signaling pathway, upregulate the expression of MMPs, accelerate type II collagen degradation, thereby increasing CTX-II levels, and forming a positive feedback loop between inflammation and cartilage destruction. Findings from this study revealed that both ELASEM^®^Flex and ELASEM^®^ProFlex significantly reduced the serum levels of CTX-II. Specifically, ELASEM^®^Flex inhibited serum levels of CTX-II at weeks 2 and 4 by 20% and 32%, respectively, while ELASEM®ProFlex achieved inhibitions of 32% and 40% at weeks 2 and 4, respectively. These results demonstrate that both ELASEM^®^Flex and ELASEM^®^ProFlex can significantly reduce the degradation of type II collagen in arthritic rats, with ELASEM^®^ProFlex exhibiting a significantly superior effect compared to ELASEM^®^Flex.

Histopathological findings further validated the therapeutic effects of ELASEM^®^Flex and ELASEM^®^ProFlex in arthritic rats. Following treatment with ELASEM^®^Flex and ELASEM^®^ProFlex, notable improvements were observed: the areas of hyaline cartilage thinning were reduced, chondrocyte proliferation and numbers increased, joint space narrowing was alleviated, the extent of synovial tissue congestion and thickening diminished, and subchondral bone tissue showed improvements. Additionally, compared with the OA model group, staining intensity for chondrocytes and cartilage matrix was enhanced. The Mankin’s scoring system is a widely recognized histopathological tool that evaluates articular cartilage damage based on criteria such as surface damage, chondrocytes, and matrix staining intensity, with higher scores indicating more severe OA progression [18]. In our study, post-treatment with ELASEM^®^Flex and ELASEM^®^ProFlex resulted in significantly lower Mankin’s scores compared to the OA model group (P < 0.05), with reductions of 20% in the ELASEM^®^Flex group (D) and 21% in the ELASEM^®^ProFlex group (E), respectively. These results confirm that ELASEM^®^Flex and ELASEM^®^ProFlex can promote cartilage repair and effectively mitigate cartilage damage in OA rats.

The results of this study are consistent with previous research. Wedekind et al. [52] showed that EM can significantly reduce serum levels of biomarkers such as IL-1β, CTX-II, cartilage oligomeric matrix protein (COMP), and α-2-macroglobulin (A2M), and improve joint inflammation in collagen-induced arthritis rats. Similarly, Sim et al. [11] confirmed in an MIA-induced rat OA model that EM can inhibit the expression of IL-1β, IL-6, matrix metalloproteinases (MMPs), etc., reduce the serum level of CTX-II, thereby alleviating cartilage damage and protecting cartilage and joint tissues. The cartilage-protective effects of EM may be mediated through the following mechanisms: First, EM exerts direct immunomodulatory effects. In vitro studies have shown that EM can inhibit the production of various pro-inflammatory cytokines (such as TNF-α, IFN-γ) in mitogen-activated human immune cells [53]. Second, EM may also exert indirect immunomodulatory effects via EM-mediated activation of NF-κB, through an oral tolerance mechanism initiated in gut-associated lymphoid tissue [54]. Third, EM can suppress inflammatory markers and extracellular matrix degrading enzymes in cartilage and synovium, while upregulating the expression of chondrogenic genes, which helps to reduce joint degeneration and synovial inflammation [18].

The strengths of this study are as follows: (1) By comparing the efficacy differences between the two EM preparations, it was found that ELASEM^®^ProFlex has more advantages in sustained analgesic effect, anti-inflammation and cartilage protection; (2) Both EM preparations exhibit good analgesic effects. Specifically, ELASEM^®^ProFlex shows significantly better analgesic efficacy than diclofenac sodium with a faster onset of action, while ELASEM^®^Flex demonstrates analgesic efficacy comparable to that of diclofenac sodium; (3) A multi-dimensional efficacy evaluation system covering MPT, proinflammatory cytokine, degradation markers, and histopathology was constructed to comprehensively evaluate the efficacy of EM; (4) Through dynamic detection of MPT, IL-1β, COX-2, TNF-α, and CTX-II, the regulatory effects of EM on collagen metabolism, arthritic pain, and inflammatory factors were quantified, providing new evidence for mechanism research. This study still has certain limitations: (1) The activity of MMPs and oxidative stress indicators in cartilage tissue were not detected, making it difficult to fully explain the matrix protection mechanism of EM; (2) The mechanism of action of EM was not explored in depth. Future research can be carried in the following directions: (1)Using proteomics technology to screen the key active components of EM and clarify its target; (2) Combining preclinical imaging technologies (such as micro-CT) to dynamically monitor changes in joint structure, providing more sufficient basis for clinical trial design.

## Conclusion

In conclusion, ELASEM^®^Flex and ELASEM^®^ProFlex can exert preventive and reparative effects on knee OA in rats by alleviating arthritis pain, inhibiting the expression of proinflammatory cytokines to reduce inflammatory responses, mitigating type II collagen degradation, and promoting chondrocyte proliferation. Among the two, ELASEM^®^ProFlex has a faster onset of action and more pronounced anti-inflammatory effects compared with ELASEM^®^Flex. Both ELASEM^®^Flex and ELASEM^®^ProFlex hold promise for development into functional foods or innovative drugs for the prevention and treatment of OA.
